# Frequency-dependence may moderate fitness costs linked to reduced bird song complexity

**DOI:** 10.1098/rsos.240766

**Published:** 2024-10-16

**Authors:** Daniel Appleby, Naomi Langmore, Robert Heinsohn, Ross Crates

**Affiliations:** ^1^ Fenner School of Environment and Society, Australian National University, Acton, Australian Capital Territory, Australia; ^2^ Research School of Biology, Australian National University, Acton, Australian Capital Territory, Australia

**Keywords:** birdsong, culture, conservation

## Abstract

Animal cultures can undergo rapid changes associated with innovations, revolutions or population decline. Where a rapid shift results in reduced complexity of cultural behaviours, it may have fitness consequences for individuals. Here, we report a dramatic shift in the dominant song type of critically endangered wild regent honeyeaters *Anthochaera phrygia*. Between 2015 and 2019, most males in the Blue Mountains sang a typical regent honeyeater song (typical Blue Mountains song), but 5%–10% sang an abbreviated version of the song with half the number of syllables (the clipped Blue Mountains song), which was associated with lower pairing success. Since 2020, the proportion of males singing the clipped Blue Mountains song has increased to 50%–75% each year. The likelihood of successful pairing in these males showed a significant concomitant increase, suggesting that the fitness costs associated with the abbreviated song decreased as it became the dominant song type. Our results suggest that the fitness consequences of loss of song complexity in declining and fragmented populations may be ameliorated by frequency-dependent shifts in song type preference.

## 1. Introduction

Animal cultures are learned behaviours, traditions or knowledge that are transmitted within a group of animals through social learning or imitation, and maintained across generations [1,2]. They encompass the shared behaviours, skills or customs that are maintained between generations, shaping the behaviour and practices of societies or populations. Cultures may aid or hinder the rate of survival [3,4], reproduction [5] or dispersal [6] for individuals within a species and as such, understanding these processes is vital for the conservation and management of threatened species.

Recent studies have shed light on instances wherein animal cultures undergo transformative changes. Research conducted by Aplin *et al.* [7] and Klump *et al.* [8] has elucidated cases of animal cultures having been subject to the influence of innovation, manifesting in the adoption of novel feeding techniques, tool use or other behavioural innovations that subsequently permeate through a population. Animal cultures may also undergo revolutionary changes [9]. For example, humpback whales *Megaptera noevaeagliae* in the South Pacific, underwent a cultural revolution, in which the population’s entire song repertoire was replaced in a period of 2 years following the introduction of novel songs sung by individuals originating from a geographically distinct population [9]. The novelty of introduced behaviours may indicate some adaptive advantages such as increased genetic diversity or other fitness traits which can rapidly be selected for [10]. Such cultural modifications could hold profound implications for the dynamics and ecological interactions within a species and its respective ecosystem.

Population decline [11] and the fragmentation of habitats [11] can also significantly alter animal cultures. Investigations conducted by Paxton *et al.* [12], Crates *et al.* [13] and Backhouse *et al.* [14] have demonstrated that dwindling animal populations may result in the loss of cultural knowledge and associated behaviours within the remaining individuals. Revolutionary changes owing to environmental or anthropogenic change have been observed in recent years. In these cases, cultural traditions may shift to facilitate exploitation of a new resource, deal with a novel threat or simply decline, where a niche is no longer suitable [15,16]. This loss of culture can impair the adaptability and resilience of the population, potentially exacerbating the rate of population decline through Allee effects [17].

In songbirds, song repertoires and dominant song types change over time [18]. This is a normal outcome of song learning and is generally not costly to individuals. However, several recent studies have revealed that when population size declines, there is often a concomitant loss of complexity in cultural traits such as birdsong [12–14,19]. Moreover, many studies have shown that female songbirds prefer males that sing complex songs and have complex repertoires [20], so it is predicted that males with reduced song complexity will be less attractive to females, reducing reproductive success and potentially impeding conservation efforts in threatened species [21,22].

Regent honeyeaters (*Anthochaera phrygia*) are a critically endangered nectarivorous songbird whose males sing a single song that is learned early in life and maintained throughout adulthood. Within the population, there are a range of song types [13] that form loose geographical dialects. It has been previously demonstrated that males singing the song type that is the cultural norm have greater reproductive success than those that sing an abbreviated version of the song [13]. However, it is unclear whether males with the simpler song type were less successful because their songs were (i) not the cultural norm, or (ii) less complex in terms of their duration, number of syllables and number of unique syllables. A recent, rapid change in song culture provides a natural experiment that facilitates comparison between complex sexual signals (complex songs) and cultural conformity. Specifically, we aim to test whether males that sing the simpler song type continue to suffer fitness costs when the simpler song type becomes the cultural norm.

## 2. Methods

### 2.1. Study species and previous song culture research

Regent honeyeaters are a medium-sized (approx. 40 g) songbird endemic to southeastern Australia with a generation time of 3.4 years, a mean lifespan of 5–6 years and a monogamous breeding strategy [23]. The population has been in significant decline from approximately 1500 [24] to less than 300 individuals over three decades [13] and is headed for extinction within a decade without enhanced conservation action [23]. Severe habitat loss is the primary driver of population decline [25].

Crates *et al.* [13] and Powys [26] identified that male regent honeyeaters’ songs have geographical song types within the two remaining New South Wales (NSW) breeding areas—the greater Blue Mountains and the Northern Tablelands. The greater Blue Mountains contains the largest remaining population of regent honeyeaters and males originating from this area that sing species-specific songs and produce one of two song types—the ‘typical Blue Mountains’ or the ‘clipped Blue Mountains’. The clipped Blue Mountains song is a simplified version of the typical Blue Mountains song with half the number of syllables [13]. Males in the Northern Tablelands that sing species-specific songs produce one distinct song, referred to as the ‘Northern Tablelands’ song type. Moreover, 12% of wild males across both locations fail to sing any version of the species-specific song and instead sing songs resembling those of other bird species- the ‘interspecific’ song type. Interspecific songs were more common in males occurring in areas of low population density. Aberrant songs (including the clipped Blue Mountains song) were associated with fitness costs in regent honeyeaters: males who sang songs that differed from the regional cultural norm were less likely to be paired with a female, and those that were paired to a female were less likely to initiate a nesting attempt [13]. Aberrant songs also occurred in zoo-bred males contributing to a reintroduction programme, which sang rudimentary songs differing from all wild song types [13].

### 2.2. Data collection

We compiled a database of all wild-born male regent honeyeaters located between 2015 and 2022. The database did not contain zoo-bred males released to the wild because of (i) the relatively small number of males resighted at least six months post-release; and (ii) the fact that the release location of zoo-bred regent honeyeaters shifted from northern Victoria to the greater Blue Mountains in the middle of our study period. We obtained data on wild males from two sources: the National Regent Honeyeater Monitoring Program—a standardized survey protocol surveying over 1200 sites throughout the species’ range each spring [13]; and a public sightings database managed by BirdLife Australia. We were able to sex birds in the field based on a combination of colour bands, size, plumage traits, singing and/or nesting behaviour. We were able to identify males individually through a combination of colour bands on a proportion of wild males (*n* = 57) and/or their partner females (*n* = 21), the unique song attributes of some males, the location of nests and/or a lack of other birds nearby at the time they were sighted. While it is possible that occasionally an unbanded male was included as separate data points in different years, colour mark resighting data suggest the overall resighting rate is less than 20% and the annual resighting rate is much lower than this. This relatively low resighting rate is likely to be explained by a combination of the highly nomadic life history of the regent honeyeater, generational turnover and population decline. Therefore, we are confident that the majority of unbanded males included in the dataset represent different individuals. Nine colour-banded males were resighted in more than one breeding season. We accounted for possible non-independence by including ‘male ID’ as a random term in the models. For each male, we calculated an index of population density by counting the number of other males sighted within 1 km and 50 km of each male in the same year. The full methodology used in this study is described by Crates *et al.* [13], incorporating an additional 3 years’ data on the wild population.

### 2.3. Song classification

Juvenile regent honeyeaters learn songs by associating with older conspecific tutors [13]. Only males produce a full song, and recent evidence from zoo-breeding experiments shows male regent honeyeaters are close-ended song learners that crystallize their adult-type song within the first nine months of life [27]. There is no evidence in the wild or zoo-bred population that individual males sing more than one song type.

**Figure 1.**
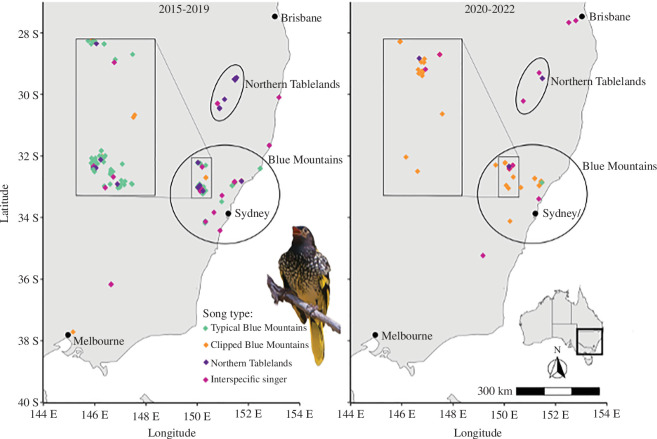
The location of wild male regent honeyeaters with colour labels indicating their song type, from 2015 to 2019 (left) and from 2020 to 2022 (right). Ellipses identify the location of the Blue Mountains and Northern Tablelands breeding areas, with the Blue Mountains key breeding areas shown at finer scale in insets. Owing to map scale, not all individuals’ records in areas of high population density are visible. Inset on right: location of study area on a national scale.

**Figure 2.**
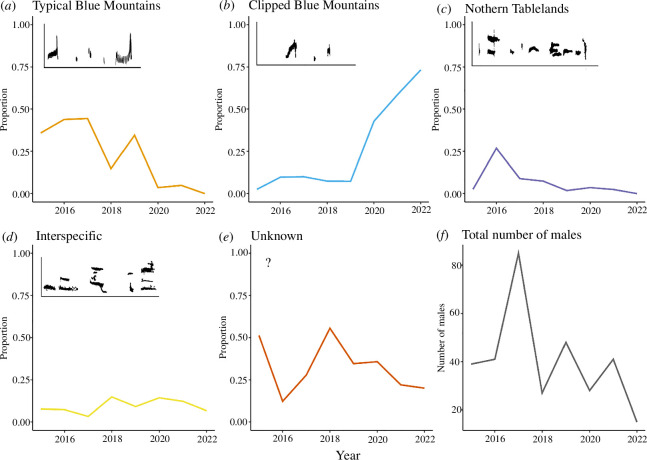
The annual proportion of wild male regent honeyeaters singing each song type by year (*a–e*); and the total number of males detected per year (*f*). Insets in (*a–d*) show representative spectrograms of each song type. The frequency on the *y*-axis is between 0 and 10 kHz, the time on the *x*-axis is variable. See the electronic supplementary material for more detailed spectrograms.

We assigned wild males’ songs to one of the following five song types ([13]; figures 1 and 2; electronic supplementary material, figure S1):

- typical Blue Mountains: the ‘regional cultural norm’ song type of wild males occurring in the greater Blue Mountains—the largest remaining wild population—during the period 2015–2019;
- clipped Blue Mountains: similar to the typical Blue Mountains song, but males singing this song type omit the final three syllables of the typical Blue Mountains song;
- typical Northern Tablelands: birds predominantly occurring in the northern NSW breeding population with a song type distinct from both Blue Mountains song types;
- interspecific singer: males that do not sing any of the above three song types and have instead learned to sing the song of one of a number of different bird species including little wattlebird *Anthochaera chrysoptera*, noisy friarbird *Philemon corniculatus*, little friarbird *Philemon citreogularis* and spiny-cheeked honeyeater *Acanthagenys rufogularis* [13]; and
- unknown: males that were located in the wild but either did not sing, their song was not recorded or was not heard by a species’ expert.

Crates *et al.* [13] used discriminant function analysis of the spectral attributes of high-quality recordings of 47 males’ songs to demonstrate the similarities within and the differences between the song types. They then used blind classification tests of song recordings by recognized species’ experts to further demonstrate the repeatability of the song classification approach. For the additional 3 years’ data used in this study, we used the same species’ experts for the classification of song types that contributed to Crates *et al.* [13] and obtained field recordings from a further 14 males (electronic supplementary material, file T1).

### 2.4. Statistical analysis

#### 2.4.1. Changes in population density and dominant song type

We first used Mann–Whitney *U*-tests to assess how the density of the wild male population differed between the 2015–2019 and 2020–2022 study periods and between the 1 km and 50 km scales. We then calculated the proportion of males singing each song type for each year. To assess the changes in the proportion of males singing each song type, we calculated the mean proportion for each song type within the periods 2015–2019 and 2020–2022. This provided values for the proportions of individuals associated with each song type in each year and within each period. To determine if there were significant differences between the proportions of males singing each song type in each period, we conducted Mann-Whitney *U*-tests using the base ‘stats’ package in R (R Core Team, 2021 [28]).

#### 2.4.2. Fitness correlates of male song type

To assess fitness associations with song type, we examined the likelihood that birds detected during the breeding season (July to January) were (i) paired or unpaired, and (ii) in paired birds, whether or not they successfully initiated a nesting attempt in which their partner female laid at least one egg. Details on nest monitoring methodology are provided in Crates *et al.* [29]. We fitted mixed-effects logistic regressions with the binomial response variables of ‘paired or unpaired’, and then, for paired birds ‘nested or failed to nest’, including song type as a fixed effect (formula: paired or not~song type + (1|male ID), family= ‘binomial’; nested or not~songtype + (1|male ID), family ‘binomial’). We tested this on both the 2015–2019 and the 2020–2022 datasets, as this was the period during which we noted a rapid shift in the dominant wild song type (see §3, results). In Crates *et al.* [13], a fitness cost was reported for birds that sang songs outside the regional cultural norm. To assess changes to this fitness cost, we repeated the logistic regressions on the 2020–2022 dataset keeping ‘typical Blue Mountains’ and ‘Northern Tablelands’ song types as the cultural norm for their respective geographical regions. We then conducted the same ‘paired’ and ‘nested’ logistic regressions on this dataset. Owing to the small sample size of birds singing ‘typical Blue Mountains’ songs in the 2020–2022 period, we conducted an additional analysis of the temporal changes in fitness correlates for birds singing the ‘clipped Blue Mountains’ song. We fitted mixed-effects logistic regressions using time period as the predictor and binomial response variables of ‘paired or unpaired’, and then, for paired birds ‘nested or failed to nest’, including period as a fixed effect (formula: paired or not~time period + (1|male ID), family= ‘binomial’; nested or not~time period + (1|male ID), family ‘binomial’). We also fitted the same models with a subset of the data including only birds whose songs were classified as the predominant song type detected during each study period (2015–2019 – ‘typical Blue Mountains’, 2020–2022 – ‘clipped Blue Mountains’).

## 3. Results

The sightings database contained 324 individual males located during the periods 2015–2019 (*n* = 241) and 2020−2022 (*n* = 75) (figure 1).

**Figure 3.**
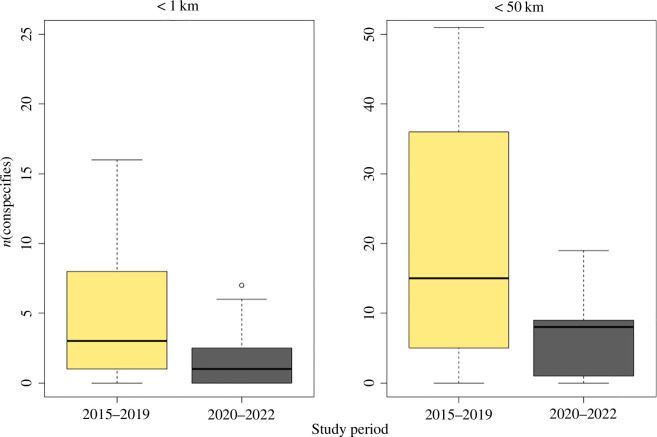
Boxplots showing the number of male regent honeyeaters detected within (*a*) 1 km; and (*b*) 50 km of each focal male in each of the two study periods (2015–2019: *n* = 252, 2020–2022: *n* = 84).

The total number of males located annually varied considerably but showed an overall downward trend (figure 2). The density of the population also decreased significantly at both the 1 km range (Mann–Whitney *U*-tests *W* = 13 465, *p <* 0.001) and at the 50 km range (*W* = 14 174, *p* < 0.001; figure 3). Thus, there was a significant decrease in the number of potential song tutors for juvenile males over time (figure 3).

The songs of 218 males were classified as belonging to one of four song types, whereas those of 106 males were either not recorded or not heard in the field by a species’ expert and were therefore classified as unknown.

### 3.1. Song types

Major changes were detected in the proportion of males singing the typical Blue Mountains and clipped Blue Mountains song types over time (figure 2). Among males with known song types, males singing the clipped Blue Mountains song type represented only 5% of the population in 2015, and this proportion remained relatively consistent until 2019 (11% of birds with identified song type). However, the proportion of males singing the clipped Blue Mountains song type had increased to 91% of birds with an identified song type by 2022. Conversely, in 2015, 73% of males with a known song type sang the typical Blue Mountains song, which also remained relatively consistent until 2019 (52% of birds with an identified song type). After 2020, the proportion of males singing the typical Blue Mountains song type had fallen to 5%. No birds were identified to be singing this song type by 2022.

The average proportion of males singing the typical Blue Mountains song type was significantly lower (*U* = 15, *p* = 0.03) in the 2020–2022 period (2.8% of birds) than in the 2015–2019 period (34.7%). The average proportion of males singing the clipped Blue Mountains song type increased significantly (*U* = 0, *p* = 0.03) in the 2020–2022 period (58%) compared with the 2015–2018 (5%). There was no significant difference in the proportion of males singing other song types between the two time periods (Northern Tablelands, *U* = 12, *p* = 0.25; interspecific, *U* = 6, *p* = 0.79; unknown, *U* = 10, *p* = 0.57; figure 2).

### 3.2. Fitness associations with song type

#### 3.2.1. Pairing and nesting by song type

**Figure 4.**
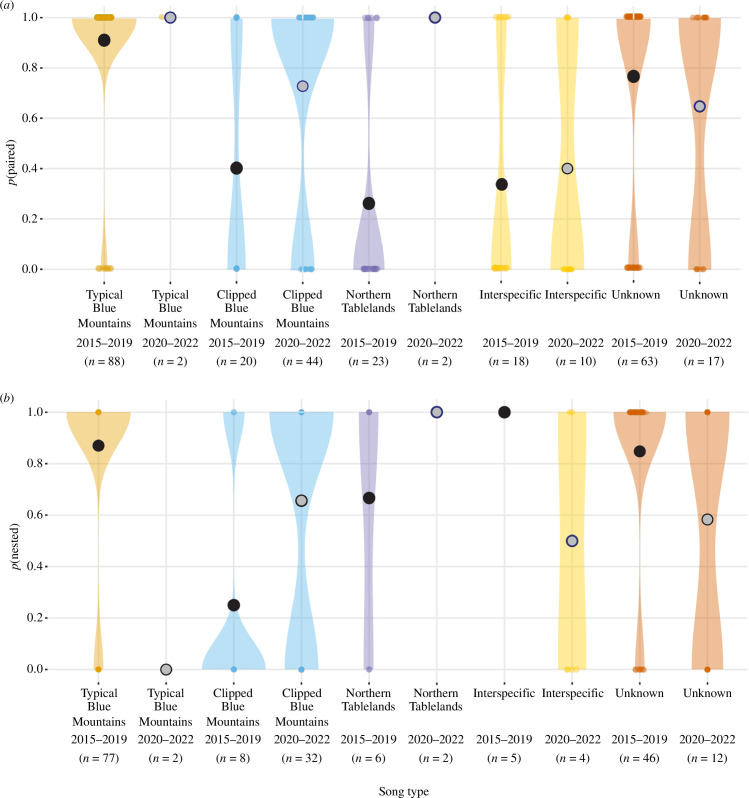
Violin plots showing the probability of wild male regent honeyeaters detected during the breeding season being (*a*) paired; or (*b*) paired males successfully initiating a nesting attempt according to song type in the periods 2015–2019 and 2020–2022. The *y*-axis shows the probability, the *x*-axis shows song type and year. The colour of the violins is according to song type, while the colour of the estimates (i.e. circles) denotes time period (black for 2015–2019, grey for 2020–2022).

During the 2015–2019 period, the probability of pairing in males singing songs other than the ‘typical Blue Mountains’ song type was significantly lower than for males that did sing this song type (table 1). However, during the 2020–2022 period, the probability of a male being paired to a female did not differ by song type. During the 2015–2019 period, the probability of pairs successfully initiating a nest was lower when the male sang the ‘clipped Blue Mountains’ song type than when the male sang the typical Blue Mountains song type. However, this difference was also not present during the 2020–2022 period (electronic supplementary material, table S1; figure 4*b*).

**Table 1.** Results of generalized linear mixed-effects models showing the relationship between the probability of being paired to a partner female, and of paired males to successfully initiate a nesting attempt and the time period. (** β** is the coefficient which describes the change in probability relative to the reference time period of 2015–2019.)

response	test	reference group (*n*)	level	*n*	β	s.e.	*Z*	*p*
paired	time period (clipped Blue Mountains only)	2015–2019 (20)	2020–2022	44	1.39	0.57	2.44	**0.02**
nested		2015–2019 (8)	2022–2022	32	1.75	0.90	1.95	**0.05**
paired	predominant song type	typical Blue Mountains and Northern Tablelands 2015–2019 (105)	2022–2022	46	−0.47	0.42	−1.11	0.266
nested		clipped Blue Mountains and Northern Tablelands 2015–2019 (83)	2022–2022	32	−1.13	0.49	−2.33	**0.02**

Bold values are those that were below the significance threshold of 0.05.

**Figure 5.**
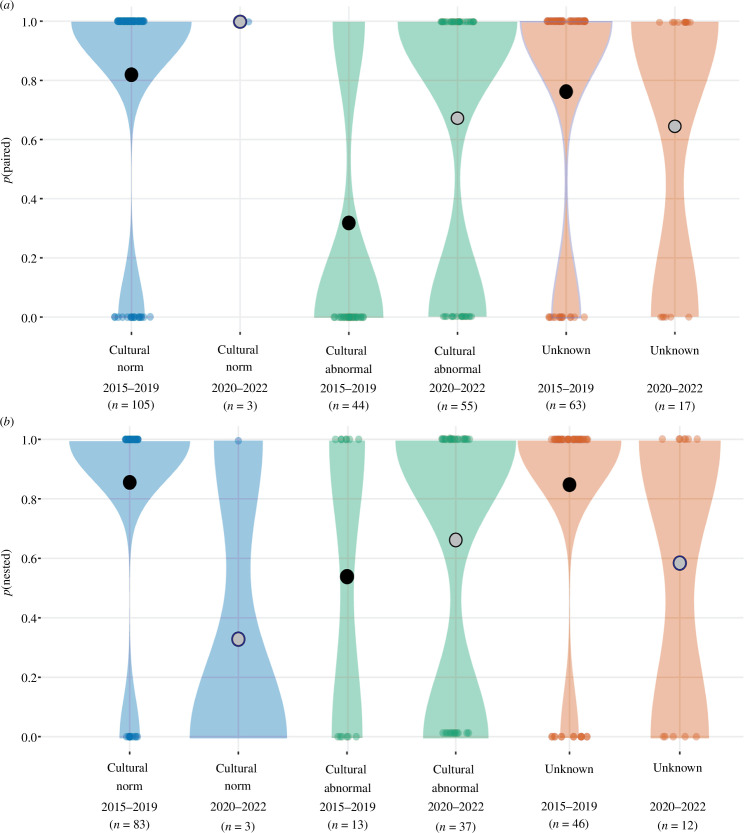
Violin plots displaying the probability of wild male regent honeyeaters detected during the breeding season being (*a*) paired; or (*b*) paired males successfully initiating a nesting attempt according to whether males sang a song type belonging to the prevailing regional cultural norm in the periods of 2015–2019 and 2020–2022. We consider the typical Blue Mountains and the Northern Tablelands song types as the cultural norm in their respective regions across both time periods. The *y*-axis shows the probability, the *x*-axis shows whether the bird’s song belonged to the regional cultural norm or not and the time period. The colour of the violins is according to cultural norm, while the colour of the predictions (i.e. circles) denotes time period (black for 2015–2019, grey for 2020–2022).

Overall, during the 2015–2019 period, there was a significantly lower probability of both nesting and pairing for males whose songs did not conform culturally (table 1). During the 2020–2022 period there was no significant difference in the probability of males that sang songs outside the cultural norm (as defined by the 2015–2019 standard) being paired or of paired males nesting (electronic supplementary material, table S1; figure 5). Despite comprehensive census of this species, its nomadic life history, cultural song shifts and continued population decline result in unavoidable small samples of males singing the typical Blue Mountains and Northern Tablelands songs during the 2020–2022 period, and should therefore be interpreted with caution. However, relative to the 2015–2019 period, the pairing and nesting success of birds singing the ‘clipped Blue Mountains’ song type increased significantly in the 2020–2022 period (table 1; figure 4). Comparison between birds singing the most common song type in each time-period (2015–2019: ‘typical Blue Mountains’; 2020–2022: ‘clipped Blue Mountains’) revealed that the probability of successfully pairing was not significantly different, however, the probability of paired birds successfully nesting was significantly lower for clipped Blue Mountains singers between 2020 and 2022 than for typical Blue Mountains singers between 2015 and 2019 (table 1).

## 4. Discussion

Between 2015 and 2022, there was a substantial decrease in both the annual number of wild male regent honeyeaters located and the density of the remaining population. Over the same period, the average proportion of males singing the typical Blue Mountains song decreased from 35% to 0%, while the proportion singing the clipped Blue Mountains song increased from 2.5% to 73.3%. The fitness costs associated with singing songs that differed from the cultural norm during the 2015–2019 period significantly decreased in 2020–2022, as the previously culturally dominant typical Blue Mountains song disappeared from the population. While unavoidably small sample sizes prevented robust comparison of the fitness correlates of contemporary birds singing ‘typical Blue Mountains’ songs with the now dominant ‘clipped Blue Mountains’ songs, comparison of contemporary birds singing ‘clipped Blue Mountains’ songs with their 2015–2019 counterparts show that the fitness cost associated with singing the abbreviated song type has decreased as that song type has become more dominant, suggesting that females become more accepting of males singing less complex songs as they become more common in the population.

### 4.1. Cultural song shifts linked to general generational turnover

Understanding the underlying mechanisms of rapid, population level changes towards a simpler song in nomadic species such as the regent honeyeater is a difficult task. We suggest that rather than a cultural revolution of song in regent honeyeaters, the observed rapid change may be caused by a combination of generational turnover and decreased population density, which in turn may influence the number and type of song tutors available to juvenile males. Although many species undergo rapid changes through innovation or cultural revolution [7], it is unlikely that these processes are responsible in the case of the observed changes to regent honeyeater song. Whereas most cultural innovations are adaptive [4,30,31]; loss of song complexity and learning the songs of a different species were associated with fitness costs in regent honeyeaters [13]. The more simplistic clipped Blue Mountains song may instead indicate a landmark in generational turnover, whereby the remaining adult birds singing the typical Blue Mountains song declined in numbers, and the emerging generation were limited in their exposure to tutors capable of singing the typical Blue Mountains song. The clipped Blue Mountains song is an abbreviated version of the typical Blue Mountains song with no new elements, so it is unlikely that innovation or immigration is driving a revolution as is often the case in cultural revolutions [9]. Although the specific details of regent honeyeater song learning are unknown in the wild, evidence from the zoo’s song tutoring programme suggests that core learning occurs within 100 days of fledging [27]. Therefore, it is unlikely that a cultural revolution, whereby adult birds switch song types, is occurring within regent honeyeaters. All available evidence combining colour-marked wild birds and wild birds recruited to the zoo-breeding programme indicate adult regent honeyeaters remain faithful to the single song type they learn as juveniles [13].

Population size, and in particular, population density are likely to be additional key factors contributing to the rapid change in the dominant regent honeyeater song type. This is because population size and density indirectly influence the structure and diversity of songs that juveniles are exposed to during crucial phases of song learning. Song complexity and diversity may emerge from the introduction of new song elements through cultural mutations, such as copying errors and innovation, as well as the transmission of new songs among dispersing individuals [12]. With fewer overall tutors, convergence on simpler songs may occur [11], especially as the proportion of adult birds (i.e. tutors for future generations) conforming to the simpler clipped Blue Mountains song increases. Regent honeyeaters breed in loose aggregations and the breeding season may represent the time at which there is the highest density of birds in a single location. Juvenile birds are likely to have the most social interactions during this period. However, regent honeyeaters are unlikely to learn from their fathers [13], as males do not sing while raising their young and actively chase juveniles from the nesting area. As such it is likely that juveniles learn from other suitable neighbours. As co-occurring clipped Blue Mountains singing male birds were significantly more likely to be single, and as such more vocal during the 2015–2019 period, it also is possible that a demonstrator bias towards the most vocal birds [32] influences the early song learning during birds’ sensory learning phase. Given that the clipped Blue Mountains song is an abbreviated version of the typical Blue Mountains song, a conformist bias [32] towards the most common syllables may reinforce song learning of the clipped Blue Mountains song through selective attrition [33].

Crates *et al.* [13] showed that a decline in the regent honeyeater population was linked to an increase in the proportion of interspecific singers, however, further decline appears not to have increased the proportion of detected interspecific singers. In this case, the number of interspecific singers may be a function of the number of isolated birds in the population, which may have remained stable or declined. In 2015–2018, the typical Blue Mountains song type represented the dominant cultural norm and was most prevalent in birds that occurred in higher densities. In the 2020–2022 period, the clipped Blue Mountains song type was prevalent in birds that occurred in higher densities, suggesting that oblique transmission occurs between other suitable adult tutors and juvenile birds.

### 4.2. Temporal fitness associations with song type

The fitness costs associated with singing the simpler, but now culturally dominant clipped Blue Mountains song type decreased during the 2020–2022 period. Given that female regent honeyeaters have been shown to prefer the songs of birds they are exposed to and familiar with as juveniles [34], this decrease may primarily reflect a change in female preference for the familiar songs of present-day males, demonstrating a concomitant frequency-dependent shift in the preferences for song types. This could also explain the prevalence of males singing both typical Blue Mountains and Northern Tablelands song types being paired. It is possible that males singing typical Blue Mountains and Northern Tablelands songs may be paired with older females with whom they have previous breeding experience or that those females experienced these now uncommon song types as a juvenile. Alternatively, this may reflect a relaxation of selective pressures on the quality of song in mate choice. As Crates *et al.* [13] posited, where mate choice is severely limited either through competition for optimal males, or as probably the case here the cost of female search time [35], the fitness costs associated with female choosiness may force females to select males with songs that do not indicate the optimum level of fitness [36]. It is evident that song type is not an absolute barrier to reproduction in regent honeyeaters [13], as some birds with even the most aberrant songs were able to successfully find a mate. In these cases, however, pairing with a non-preferred mate may carry a fitness cost in its own right. The most direct mate competition between dialects occurred in the 2015–2019 period where a choice between males singing typical Blue Mountains and clipped Blue Mountains songs was most available. During this period, paired clipped Blue Mountains birds only proceeded to build a nest 25% of the time, this may be because of females being paired with ‘unattractive’ mates. This is consistent with findings from Griffith *et al.* [36]; where mate choice was constrained to non-preferred mates, female Gouldian finches (*Erythrura gouldiae*) had higher levels of corticosterone and delayed egg-laying relative to females that were paired with preferred mates. During the 2020–2022 period, paired clipped Blue Mountains singers proceeded to nesting 62.5% of the time. This may indicate that clipped Blue Mountains songs are at least neutral in terms of preference. However, the probability of birds singing the now dominant clipped Blue Mountains song type being paired is lower than that of birds singing the typical Blue Mountains song type for the 2015–2019 period. This could be owing to an increasingly male-biased wild population [29], as a lack of adult females is a pervasive problem in many endangered populations [37].

The presence of Allee effects can impose fitness costs on the population [38], and may arise in regent honeyeaters from reduced communication and the loss of critical cultural knowledge within their unnaturally small and sparsely distributed population. These effects may decrease cooperation and diminish the transmission of important behaviours. Therefore, while the fitness costs for birds singing the now dominant, but simpler clipped Blue Mountains song may have diminished—potentially owing to females selecting mates that conform to the new cultural norm—the decrease in population at large might still cause further loss of critical cultural information and impose potential fitness costs.

Previous studies on song complexity in birds show that song complexity may predict the viability of small, fragmented populations [19]. On the contrary, our results suggest that loss of song complexity entailed an initial decrease in reproductive success, but as the simpler songs became the cultural norm there was some increase in reproductive success in males singing the simpler song, suggesting that populations may adjust to loss of some forms of cultural complexity.

## Data Availability

Data used in this study has been uploaded to the Dryad data repository [39]. Supplementary material is available online [40].
